# Neglected outcomes of cerebrovascular disorders in neuromuscular diseases

**DOI:** 10.1111/ene.16569

**Published:** 2024-11-27

**Authors:** Benedikt Schoser

**Affiliations:** ^1^ Friedrich‐Baur‐Institute Department of Neurology LMU Clinics Munich Germany

Stroke and multisystemic features are easily overlooked in neuromuscular disorders (NMDs). The scientific literature is limited to this topic, and long‐term outcomes are rarely reported. In this issue, Al‐Salahat and colleagues report on cerebrovascular diseases (CVDs) outcomes in patients with muscular dystrophies (MD). A 10‐year screening in their nationwide readmission database identified hospitalizations for CVDs, including ischemic stroke, intracerebral haemorrhage, subarachnoid haemorrhage, and other unspecified CVDs.

They found in 6,129,132 CVD hospitalizations, that 0.03% (2098) were MD patients. Ischemic stroke was the most common type, followed by intracerebral hemorrhage. MD patients had 41% lower odds of routine discharge, 9% greater costs, 30% lower odds of 90‐day all‐cause readmissions, and a higher in‐patient mortality [1]. These results point clearly towards a higher risk of scarce outcomes of CVD in MD patients. This emphasizes a high need for boosted access to amended post‐discharge care, like stroke rehabilitation for people living with MD.

Therefore, the authors presented an essential work for the overall health of neuromuscular patients. To widen the scope of this report, considering stroke sequelae and outcomes in Duchenne muscular dystrophy, the most frequent x‐linked MD in young adults, one report, covering a total observation period of 400 patient‐years, identified four DMD with juvenile arterial ischemic strokes. Systemic thrombolysis in two patients resulted in a regression of stroke symptoms, but one patient died of septic pneumonia and cardiac failure 24 days after thrombolysis. Both other patients persisted with severe infarction‐related symptoms, with an early death in one of them. DMD‐associated cardiomyopathy without evidence of atrial fibrillation was the only risk factor for ischemic stroke in all patients [2].

Looking at metabolic lysosomal neuromuscular disorders, Fabry disease has an esteemed birth prevalence ranging from 1 in 476,000 to 1 in 117,000. However, for stroke neurologists, recognizing the X‐linked lysosomal storage disorder of glycosphingolipid metabolism is still challenging. According to FD registry data, up to 15% of patients may experience a stroke [3]. Cerebrovascular manifestations of FD may include thrombosis, transient ischemic attacks, basilar artery ischemia and aneurysm, seizures, hemiplegia, hemianesthesia, aphasia, labyrinthine disorders, or cerebral hemorrhage. Individuals living with FD may have increased basilar artery mean diameter and linear length compared with controls. White matter lesions are frequently found on brain MRIs of FD; age and prior stroke independently predicted the burden of white matter hyperintensities [3]. Similarly, in another lysosomal storage disorder, Pompe disease, stroke‐related sequelae of megadolichobasliaris, stroke, and subarachnoidal hemorrhage can occur [4, 5, 6].

Finally, stroke‐like episodes as paroxysmal neurological sequelae may be part of specific mitochondrial diseases. It is essential to differentiate stroke‐like episodes from cerebral infarction or intracerebral hemorrhage, mainly due to the variety in management. Focal‐onset seizures, encephalopathy, and visual disturbances are prominent findings associated with stroke‐like episodes, with a preference for the posterior cerebral cortex resulting in progressive brain atrophy and dementia. The most common cause of stroke‐like episodes is the m.3243A > G variant in MT‐TL1 gene, resulting in mitochondrial myopathy, encephalopathy, lactacidosis, and stroke (MELAS). Seizure management is warranted [7, 8].

In conclusion, stroke and other cerebrovascular disorders in younger patients should alert stroke neurologists to search and screen for a genetic (neuromuscular) background. For improvement of the overall stroke prognosis in NMDs, personalized care needs to be better defined.

## AUTHOR CONTRIBUTIONS

**Benedikt Schoser:** Conceptualization; writing – review and editing; data curation; project administration; validation; writing – original draft; investigation; formal analysis; supervision; resources.

## CONFLICT OF INTEREST STATEMENT

The author reports no conflict of interest.

## Data Availability

Data sharing is not applicable to this article as no new data were created or analyzed in this study.

## References

[1] Al‐Salahat A , Dilsaver DB , Bawaneh S , Chen Y‐T . Outcomes of cerebrovascular disease in muscular dystrophies: a propensity‐matched Nationwide analysis. Eur J Neurol. 2024. 10.1111/ene.16554 PMC11625959 39575848

[2] Mehta A , Hughes DA . Fabry disease. In: Adam MP , Feldman J , Mirzaa GM , Pagon RA , Wallace SE , Amemiya A , eds. GeneReviews®. University of Washington, Seattle; 2002:1993‐2024. 20301469

[3] Schüller A , Wenninger S , Strigl‐Pill N , Schoser B . Toward deconstructing the phenotype of late‐onset Pompe disease. Am J Med Genet C Semin Med Genet. 2012;160C(1):80‐88. doi:10.1002/ajmg.c.31322 22253010

[4] Montagnese F , Granata F , Musumeci O , et al. Intracranial arterial abnormalities in patients with late onset Pompe disease (LOPD). J Inherit Metab Dis. 2016;39(3):391‐398. doi:10.1007/s10545-015-9913-x 26830551

[5] Mormina E , Musumeci O , Tessitore A , et al. Intracranial aneurysm management in patients with late‐onset Pompe disease (LOPD). Neurol Sci. 2021;42(6):2411‐2419. doi:10.1007/s10072-020-04819-2 33067680

[6] Zhao Y , Yu X , Li D , et al. Intracranial vasculopathy: an important organ damage in young adult patients with late‐onset Pompe disease. Orphanet J Rare Dis. 2024;19(1):267. doi:10.1186/s13023-024-03282-y 39010129 PMC11250947

[7] Ng YS , Gorman GS . Stroke‐like episodes in adult mitochondrial disease. Handb Clin Neurol. 2023;194:65‐78. doi:10.1016/B978-0-12-821751-1.00005-1 36813321

[8] Będkowska N , Zontek A , Paprocka J . Stroke‐like episodes in inherited neurometabolic disorders. Meta. 2022;12(10):929. doi:10.3390/metabo12100929 PMC9611026 36295831

